# ﻿A new species of *Gammarus* (Crustacea, Amphipoda, Gammaridae) from South Korea

**DOI:** 10.3897/zookeys.1117.89610

**Published:** 2022-08-11

**Authors:** Yong-Uk Ahn, Chi-Woo Lee, Gi-Sik Min

**Affiliations:** 1 Department of Biological Sciences and Bioengineering, Inha University, Incheon 22212, Republic of Korea Inha University Incheon Republic of Korea; 2 Nakdonggang National Institute of Biological Resources, Sangju 37242, Republic of Korea Nakdonggang National Institute of Biological Resources Sangju Republic of Korea

**Keywords:** COI, freshwater, gammarid, Korea, morphology, new species, taxonomy

## Abstract

A new species of freshwater gammarid, *Gammarussomaemulensis***sp. nov.**, was collected from Somaemuldo Island, South Korea. This new species is morphologically characterised by the absence of calceoli in antenna 2, absence of anteroproximal setae on bases in pereopods 6 and 7, outer ramus in uropod 3 with plumose setae on both margins, and a small terminal article of the outer ramus, which is shorter than adjacent spines. A description of the new species and morphological differences from related species are provided in the text. The new species was also compared to related species using partial sequences of the mitochondrial cytochrome *c* oxidase subunit I (COI) gene. Genetic distances of COI sequences between the new species and related species, consisting of 21.5–26.3% difference, support *Gammarussomaemulensis***sp. nov.** as a valid species. Additionally, a key to identifying *Gammarus* species in South Korea is provided.

## ﻿Introduction

The genus *Gammarus* Fabricius, 1775 is one of the most speciose genera of Amphipoda, comprising more than 200 species (Väinölä et al. 2008). *Gammarus* inhabit various environments across the Northern Hemisphere, including freshwater, brackish and littoral marine waters, and 80% of these species inhabit freshwater (Väinölä et al. 2008; Hou et al. 2018). The freshwater *Gammarus* is an essential component of freshwater ecosystems and is often used as a bioindicator for water quality assessment (Gerhardt et al. 2011). However, it is well known that morphological identification of species in this genus is difficult because of the high occurrence of convergent characteristics (Karaman and Pinkster 1977).

Freshwater *Gammarus* was first reported in Korea by Uéno (1940). Since then, 11 species of freshwater *Gammarus* have been reported and described in South Korea: *G.baengnyeongensis* Kwon, Kim, Heo & Kim, 2020; *G.gageoensis* Kim, Lee & Min, 2010; *G.galgosensis* Lee & Kim, 1980; *G.hoonsooi* Lee, 1986; *G.kyonggiensis* Lee & Seo, 1990; *G.longisaeta* Lee & Seo, 1992; *G.odaensis* Lee & Kim, 1980; *G.sobaegensis* Uéno, 1966; *G.soyoensis* Lee & Kim, 1980; *G.wangbangensis* Lee & Seo, 1992; and *G.zeongogensis* Lee & Kim, 1980 (Uéno 1940, 1966; Lee and Kim 1980; Lee 1986; Lee and Seo 1990; 1992; Kim et al. 2010; Kwon et al. 2020). Islands are known to have high levels of endemism due to geographic isolation and limited interchange with mainland biota (Whittaker and Fernández-Palacios 2007), three *Gammarus* species (*G.baengnyeongensis*, *G.gageoensis*, and *G.galgosensis*) are already known to be endemic to certain islands of South Korea. The coast of South Korea is composed of thousands of islands, thus further species diversity of the genus *Gammarus* is expected in islands of South Korea.

In the present study, a new species of the genus *Gammarus*, collected from the island of South Korea is described, based on morphological differences and mitochondrial cytochrome *c* oxidase subunit I (COI) gene sequence analyses. In addition, a key to species of *Gammarus* in South Korea is provided.

## ﻿Materials and methods

### ﻿Sampling and morphological observations

**Figure 1.**
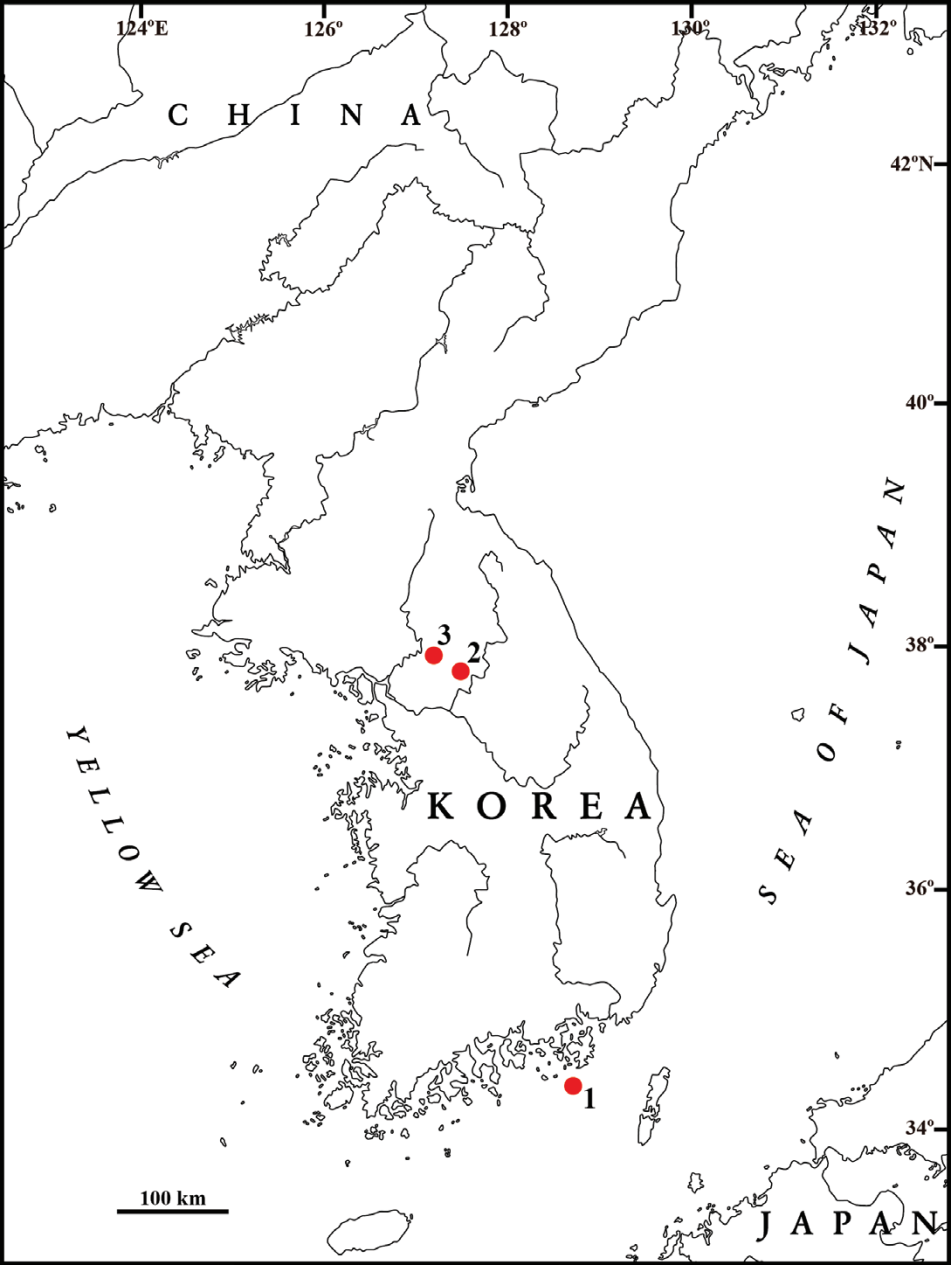
Sampling localities of *Gammarus* specimens for this study **1***Gammarussomaemulensis* sp. nov. **2***G.wangbangensis***3***G.soyoensis*. The details of localities are shown in Table 1.

*Gammarus* specimens were collected using hand-nets from three localities in South Korea (Fig. 1). The collected specimens were immediately fixed in 95% ethanol and deposited in a -20 °C refrigerator. Body length was measured along the dorsal margin of the body from the base of fist antenna to the base of the telson. Specimens were dissected under a stereomicroscope (SZX12, Olympus, Japan). All dissected appendages were mounted with glycerol on microscope slides and drawn using an optical microscope (DM2500, Leica, Germany) equipped with a drawing tube. The terminology of the setae in article 3 of mandibular palp followed Cole (1980). All the specimens were deposited at the Nakdonggang National Institute of Biological Resources (**NNIBR**), South Korea.

### ﻿Molecular data

Genomic DNA was extracted from muscle tissue of abdomen using LaboPass Tissue Mini Kit (Cosmo Genetech, Seoul, South Korea), according to the manufacturer’s instructions. COI sequences were obtained using the primer sets, LCO1490-JJ (5’-TAYTCHACYAAYCAYAAAGAYATYGG-3’) and HCO2198-JJ (5’-AWACTTCVGGRTGVCCAAARAATCA-3’) (Astrin and Stüben 2011). Polymerase chain reaction amplification was performed under the following conditions: initial denaturation at 98 °C for 1 min, followed by 5 cycles of 10 s at 98 °C, 30 s at 43 °C, and 60 s at 72 ° C. This was followed by 30 cycles of 10 s at 98 °C, 60 s at 48 °C, 60 s at 72 °C, and a 5 min extension at 72 °C. The obtained sequences were aligned using Geneious 8.1.9 (Biomatters Ltd., Auckland, New Zealand). The uncorrected *p*-distance of COI sequences was calculated using MEGA X (Kumar et al. 2018). The details of the sequences obtained in this study and those downloaded from GenBank are listed in Table 1.

**Table 1.** Species information and GenBank accession numbers used in this study.

Species	Locality (numbers in parentheses are those in Fig. 1)	Coordinates	COI	Reference
*Gammarussomaemulensis* sp. nov.	Somaemuldo-Island, Hansan-myeon, Tongyeong-si, South Korea (1)	34°37'23"N, 128°32'57.1"E	ON980527–ON980532	This study
*G.wangbangensis*	Kiji-ri, sinbuk-myeon, Pocheon-si, South Korea (2)	37°54'58"N, 127°14'9.4"E	ON980560	This study
*G.soyoensis*	Sangbongam-dong, Dongducheon-si, South Korea (3)	37°56'39.5"N, 127°5'17"E	ON980559	This study
*G.sobaegensis*	Sannae-myeon, Namwon-si, South Korea	35°53'28"N, 127°47'24"E	AB893337	Tomikawa et al. 2014
*G.baengnyeongensis*	Baengnyeongdo Island, Baengnyeong-myeon, Incheon, South Korea	37°55'37.5"N, 128°38'33.8"E	MW291608	Kwon et al. 2020
*G.zeongogensis*	Cheongsan-myeon, Yeoncheon-gun, Gyeonggi-do, South Korea	38°00'46"N, 127°07'35"E	MW353844	Kwon et al. 2020
*G.gageoensis*	Gageodo Island, Heuksan-myeon, Jeollanam-do, South Korea	34°03'N, 125°07'E	GU270652	Kim et al. 2010

## ﻿Results

**Order Amphipoda Latreille, 1816**

**Family Gammaridae Leach, 1814**

### ﻿Genus *Gammarus* Fabricius, 1775

#### ﻿ Gammarus somaemulensis sp. nov.

Taxon classificationAnimaliaAmphipodaGammaridae

7F486FC6-B28B-598E-83A4-C8C1DA2EF024

http://zoobank.org/8374BC9A-4008-4BAA-A20C-47552C679353

**Figure 2.**
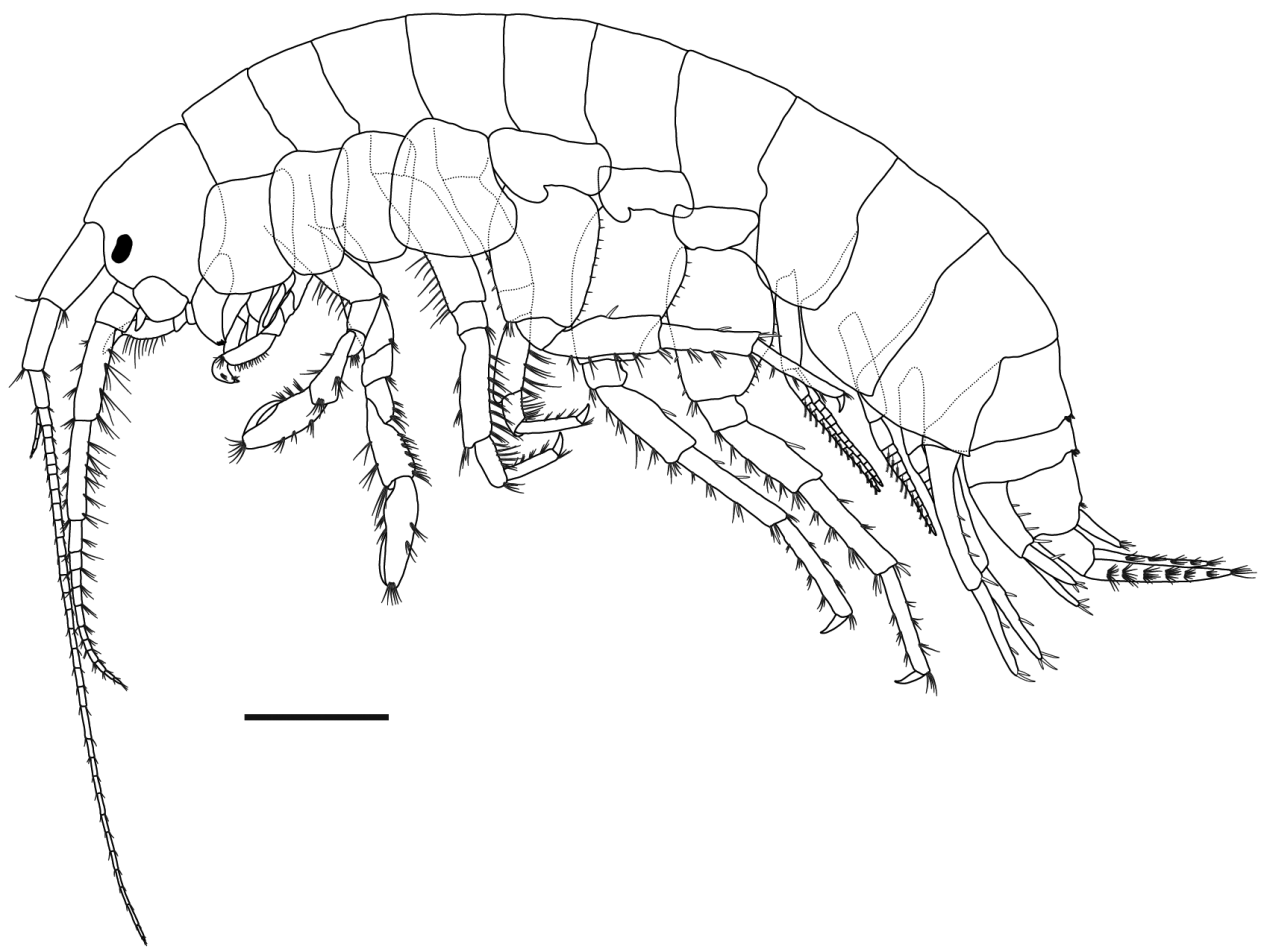
*Gammarussomaemulensis* sp. nov., male, paratype (NNIBRIV92298), habitus. Scale bar: 1.0 mm.

**Figure 3.**
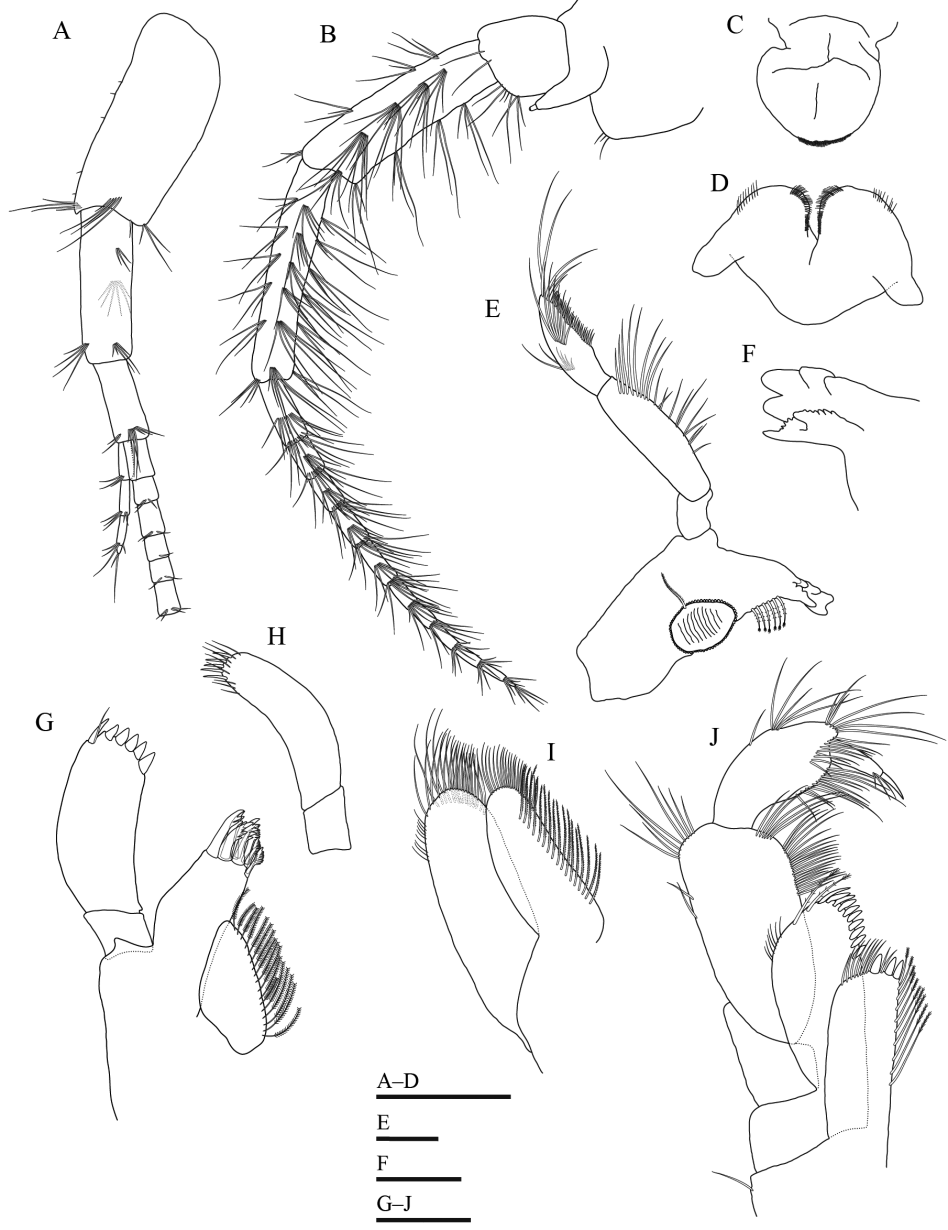
*Gammarussomaemulensis* sp. nov., male, holotype (NNIBRIV92290) **A** antenna 1, omitted from main flagellar article 7 **B** antenna 2 **C** upper lip **D** lower lip **E** left mandible **F** incisor and lacinia mobilis of right mandible **G** right maxilla 1 **H** palp of left maxilla 1 **I** maxilla 2 **J** maxilliped. Scale bars: 0.5 mm (**A–D**); 0.2 mm (**E, G–J**); 0.1mm (**F**).

**Figure 4.**
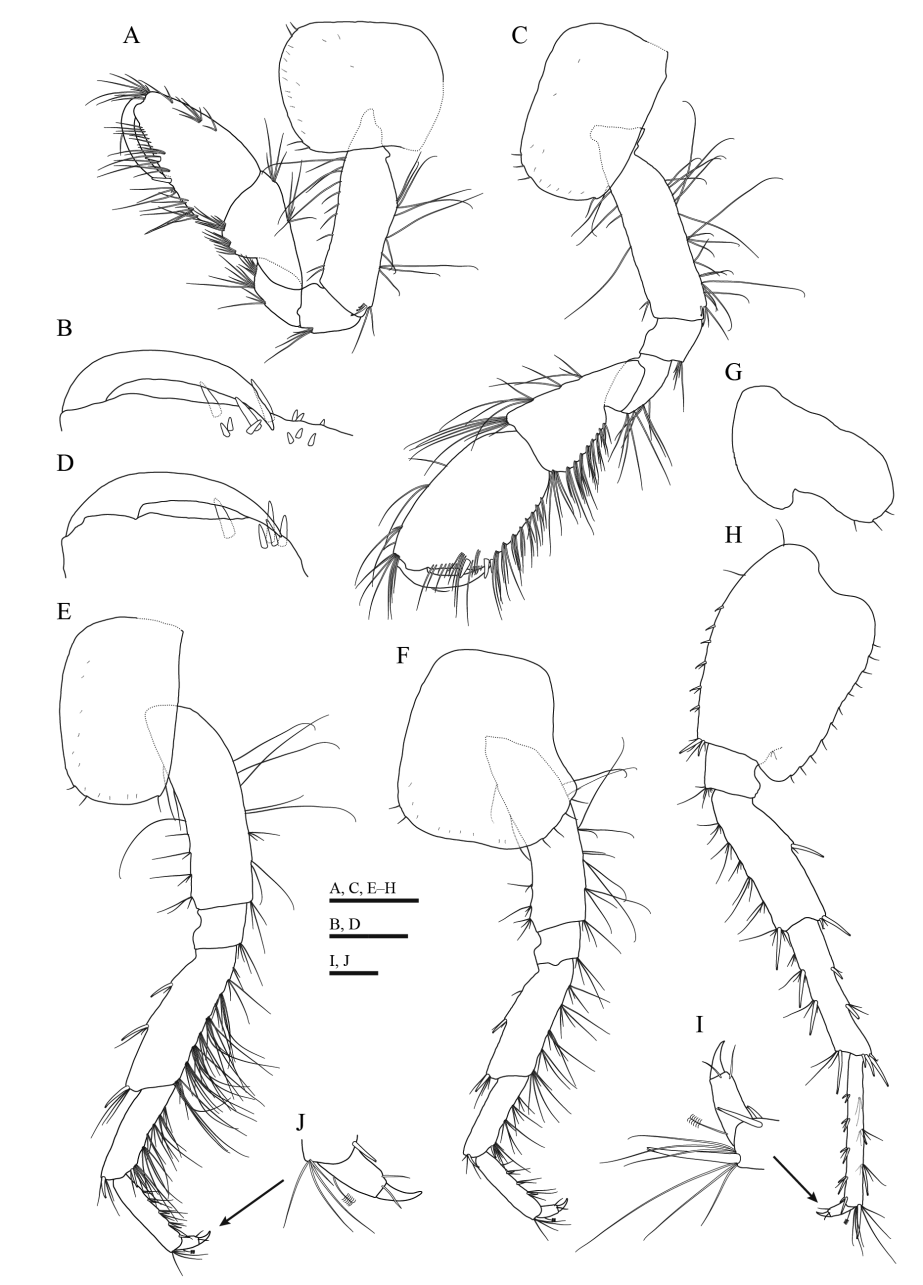
*Gammarussomaemulensis* sp. nov., male, holotype (NNIBRIV92290) **A** gnathopod 1 **B** palm of propodus and dactylus in gnathopod 1, setae omitted **C** gnathopod 2 **D** palm of propodus and dactylus in gnathopod 2, setae omitted **E** pereopod 3 **F** pereopod 4 **G** coxal plate of pereopod 5 **H** basis to dactylus of pereopod 5 **I** dactylus of pereopod 5 **J** dactylus of pereopod 3. Scale bars: 0.5 mm (**A, C, E–H**); 0.2 mm (**B, D**); 0.1 mm (**I, J**).

**Figure 5.**
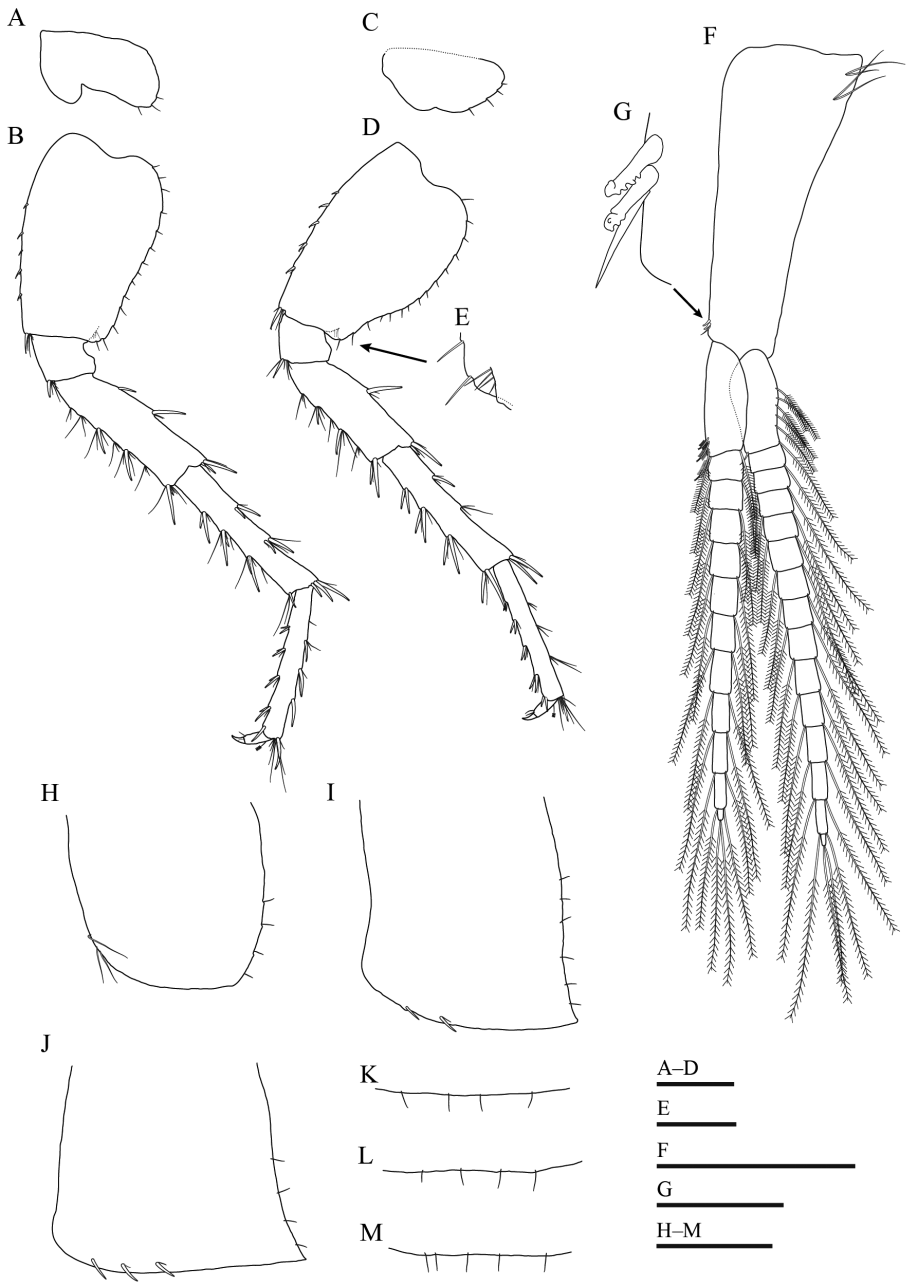
*Gammarussomaemulensis* sp. nov., male, holotype (NNIBRIV92290) **A** coxal pate of pereopod 6 **B** basis to dactylus of pereopod 6 **C** coxal pate of pereopod 7 **D** basis to dactylus of pereopod 7 **E** inner surface near posterodistal corner of basis in pereopod 7 **F** pleopod 1 **G** inner distal corner of peduncle in pleopod 1 **H–J** epimeral plates 1–3, respectively **K–M** pleonites 1–3, respectively. Scale bars: 0.5 mm (**A–D, F, H–M**); 0.2 mm (**E**); 0.05 mm (**G**).

**Figure 6.**
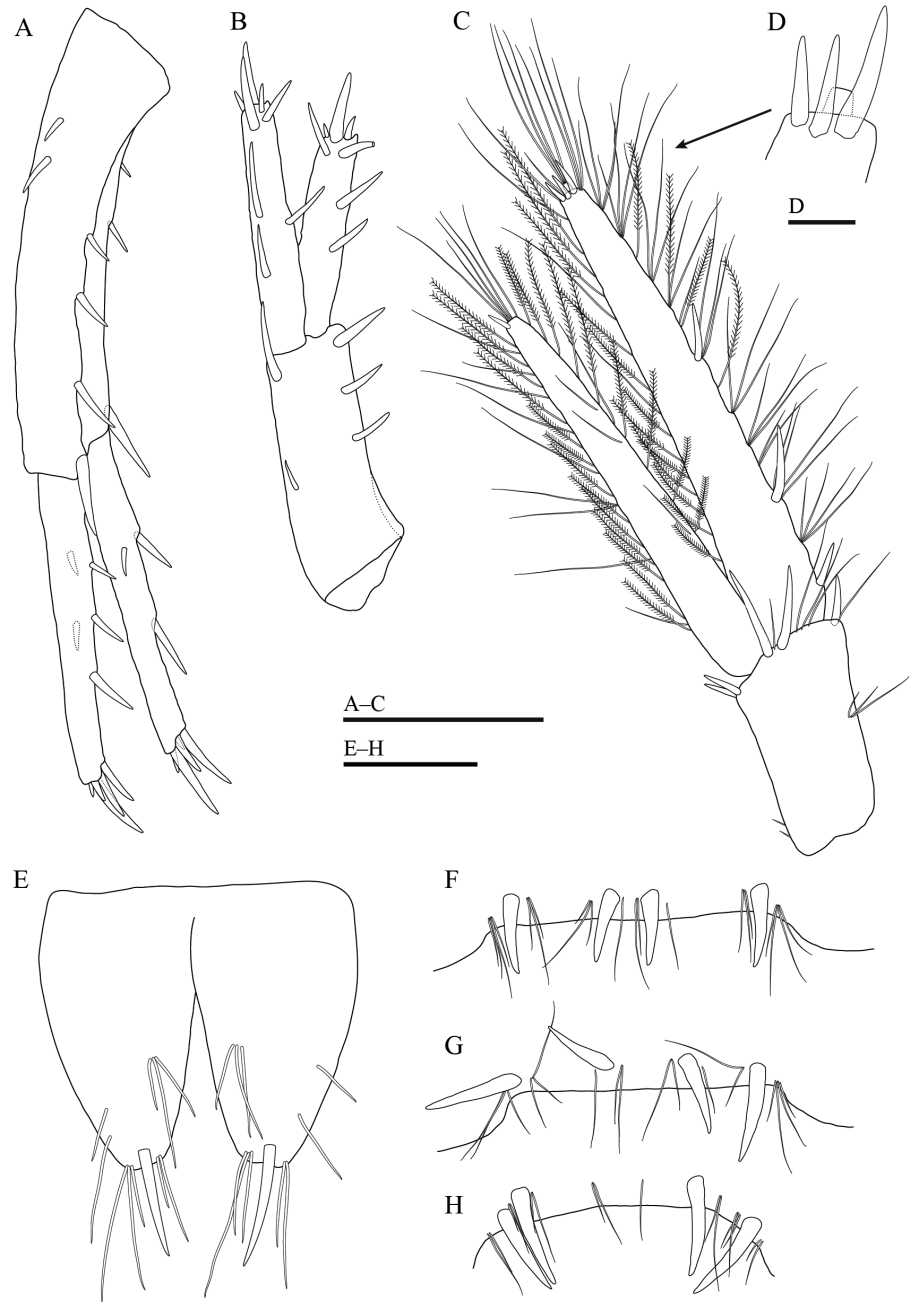
*Gammarussomaemulensis* sp. nov., male, holotype (NNIBRIV92290) **A** uropod 1 **B** uropod 2 **C** uropod 3 **D** terminal article of outer ramus in uropod 3, distal setae omitted **E** telson **F–H** urosomites 1–3, respectively. Scale bars: 0.5 mm (**A–C**); 0.05 mm (**D**); 0.2 mm (**E–H**).

**Figure 7.**
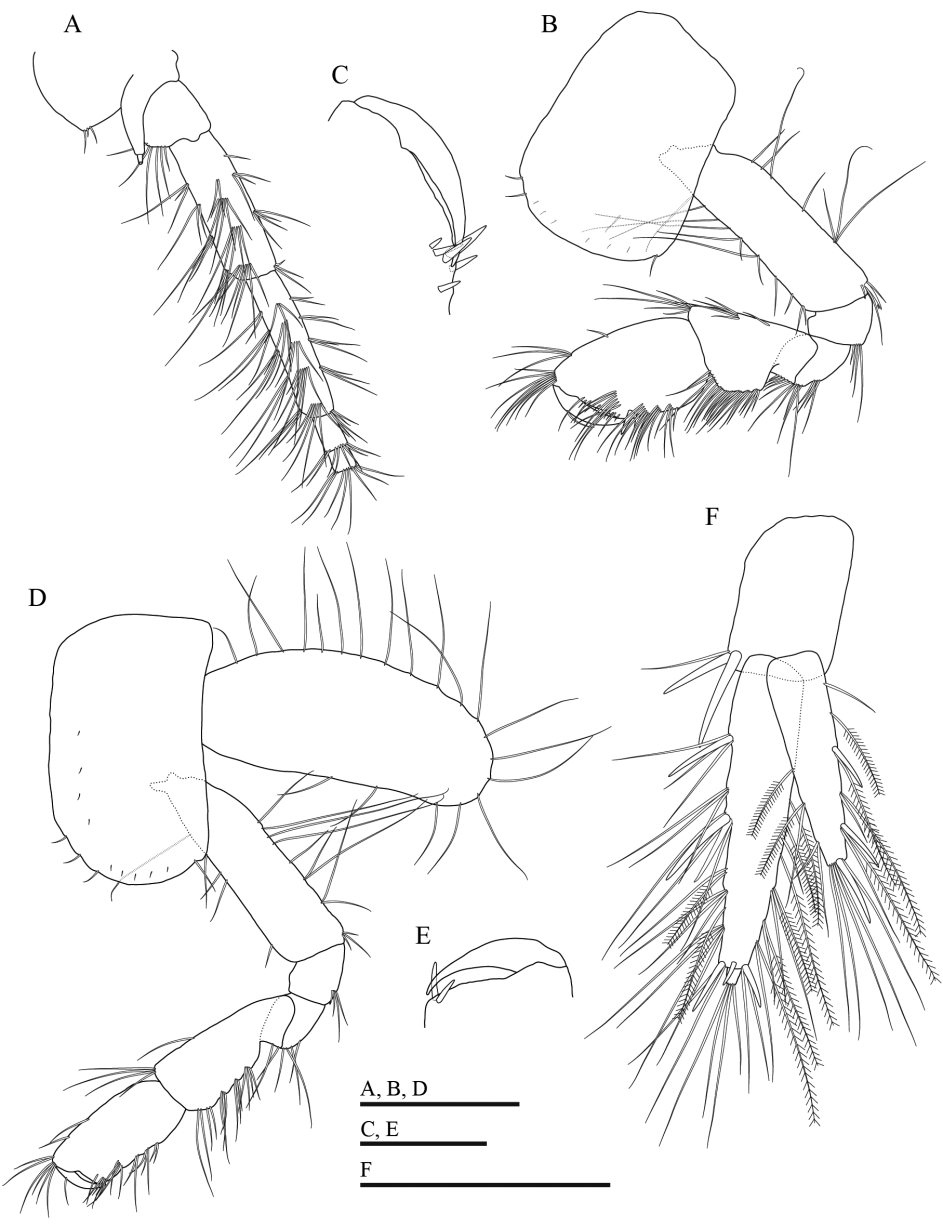
*Gammarussomaemulensis* sp. nov., female, paratype (NNIBRIV92296) **A** antenna 2, omitted from flagellar article 3 **B** gnathopod 1 **C** palm of propodus and dactylus in gnathopod 1, setae omitted **D** gnathopod 2 **E** palm of propodus and dactylus in gnathopod 2, setae omitted **F** uropod 3. Scale bars: 0.5 mm (**A, B, D, F**); 0.2 mm (**C, E**).

Figs 2 , 3 , 4 , 5 , 6 , 7 New Korean name: so-mae-mul-yeop-sae-u

##### Material examined.

***Holotype***: male, dissected on 14 slides (NNIBRIV92290), 9.0 mm, Maejuk-ri (34°37'23"N, 128°32'57.1"E), Somaemuldo Island, Hansan-myeon, Tongyeong-si, Gyeongsangnam-do, South Korea, October 7, 2021, collected by Y. U. Ahn. ***Paratypes***: male, dissected on 10 slides (NNIBRIV92291), 8.4 mm; male, dissected on 10 slides (NNIBRIV92292), 8.8 mm; male, dissected on 10 slides (NNIBRIV92293), 8.2 mm; male, dissected on 11 slides (NNIBRIV92294), 8.4 mm; male, dissected on 11 slides (NNIBRIV92295), 8.9 mm; female, dissected on 10 slides (NNIBRIV92296), 8.2 mm; female, dissected on 10 slides (NNIBRIV92297), 7.8 mm; 11 males and 3 females in ethanol vials (NNIBRIV92298–NNIBRIV92311); all other data same as holotype.

##### Etymology.

The specific name *somaemulensis* is derived from the name of the type locality, Somaemuldo Island.

##### Diagnosis.

Antenna 2 with four clusters of long setae on posterior margin of peduncular article 4, calceoli absent; pereopods 3 and 4 with long straight setae on posterior margins of merus and carpus; pereopods 6 and 7 without anteroproximal setae on basis; inner ramus of uropod 3 reaching approximately 0.8 × as long as outer ramus, outer ramus with plumose setae on both margins, terminal article of outer ramus shorter than adjacent spines.

##### Description of male.

***Head*** (Fig. 2): rostrum short; inferior antennal sinus deep; eyes reniform.

***Antenna 1*** (Fig. 3A): peduncular articles 1–3 in length ratio 1.0: 0.7: 0.4, bearing distal setae clusters on each peduncular article; main flagellum 33-articulate, each article with short distal setae; accessory flagellum four-articulate, article 4 very short.

***Antenna 2*** (Fig. 3B): peduncular article 1 with three short setae distally; gland cone tapering distally; anterior, posterior and interior margins of peduncular article 4 with four, four and five clusters of setae, respectively, length of longest seta on posterior margin 1.6 × the width of peduncular article 4; peduncular article 5 slightly longer than article 4, anterior, posterior and interior margins with six clusters of setae, respectively, length of longest seta on posterior margin 2.3 × the width of peduncular article 4; flagellum 11-articulate, calceoli absent.

***Upper lip*** (Fig. 3C): rounded, ventral margin with numerous minute setae.

***Lower lip*** (Fig. 3D): inner lobes absent, outer lobes broad.

***Mandible*** (Fig. 3E, F): incisor of left mandible with five teeth; lacinia mobilis of left mandible with four teeth; molar triturative, bearing one plumose seta; palp three-articulate in length ratio 1.0: 3.1: 2.2, article 1 unarmed, article 2 with 19 marginal setae, article 3 bearing eight B-setae on inner surface, six A-setae on outer surface, 28 D-setae on posterior margin and five E-setae apically; right mandible incisor with four teeth; lacinia mobilis of right mandible bifurcate, with small teeth.

***Maxilla 1*** (Fig. 3G, H): inner plate with 17 plumose setae; outer plate with 11 serrated spines apically; palp two-articulate and asymmetrical, right palp shorter and stouter than left palp, article 2 of right palp with five stout spines, one slender spine and one seta apically; article 2 of left palp with five slender spines and eight setae apically.

***Maxilla 2*** (Fig. 3I): inner plate bearing 17 plumose setae in an oblique row; outer plate broader than inner plate; both plates with numerous long setae apically.

***Maxilliped*** (Fig. 3J): inner plate bearing three stout spines apically; outer plate with a row of blade-like spines and two plumose setae; palp four-articulate, article 1 unarmed, inner margin of article 2 with numerous setae, article 3 curved, with numerous setae on posterior margin and a row of subapical setae, article 4 hooked, with three setae at hinge of unguis.

***Gnathopod 1*** (Fig. 4A, B): coxal plate with two setae on both anterodistal and posterodistal corners; basis with long setae on both anterior and posterior margins; length of carpus 1.4 × as long as width, 0.8 × as long as propodus, bearing two clusters of setae on anterior margin; propodus pyriform in shape, palm oblique, with one medial palmar spine and 11 spines on posterior margin; dactylus exceeding near half of propodus, outer margin with one seta.

***Gnathopod 2*** (Fig. 4C, D): coxal plate with three setae on anterodistal corner and one seta on posterodistal corner; basis similar to that of gnathopod 1; length of carpus 1.7 × as long as width, 0.8 × the length of propodus, with four clusters of setae on anterior margin; propodus subrectangular in shape, palm concave, with one medial palmar spine and four spines on posterodistal corner; dactylus curved beyond the palmar margin, bearing one seta on outer margin.

***Pereopod 3*** (Fig. 4E, J): coxal plate with two setae on anterodistal corner and one seta on posterodistal corner; basis with long setae on both anterior and posterior margins; merus bearing two spines accompanied by setae on anterior margin, eight clusters of long straight setae on posterior margin, the longest seta of them approximately 2.0 × as long as width of merus, anterodistal corner bearing one spine accompanied by setae; carpus with five clusters of long straight setae on posterior margin, one spine accompanied by setae on both anterodistal and posterodistal corners; propodus with three spines accompanied by clusters of setae on posterior margin, one spine on posterodistal corner; dactylus bearing one plumose seta on anterior margin, two setae at hinge of unguis.

***Pereopod 4*** (Fig. 4F): coxal plate with posterior excavation, bearing two setae on anterodistal corner and four setae on posterior margin; basis similar to that of pereopod 3; merus with one spine accompanied by setae on anterior margin, four clusters of long straight setae on posterior margin, the longest seta of them approximately 1.4 × as long as width of merus, anterodistal corner bearing one spine accompanied by setae; carpus with three clusters of long straight setae on posterior margin, one spine accompanied by setae on both anterodistal and posterodistal corners; propodus with three spines accompanied by clusters of setae on posterior margin; dactylus similar to that of pereopod 3.

***Pereopod 5*** (Fig. 4G–I): coxal plate bilobed, posterior lobe with three setae on posterior margin; basis with two anteroproximal setae and six small spines on anterior margin, anterodistal corner bearing two spines accompanied by setae, posterior margin with 11 short setae, posterodistal lobe developed; merus with five clusters of setae on anterior margin, one spine on posterior margin, one and two spines accompanied by setae on anterodistal and posterodistal corners, respectively; carpus with three clusters of setae and two spines on anterior margin, two spines accompanied by setae on posterior margin; propodus with four groups of spines accompanied by setae on anterior margin; dactylus bearing one plumose on posterior margin, two setae at hinge of unguis.

***Pereopod 6*** (Fig. 5A, B): coxal plate bilobed, posterior lobe with three setae on posterior margin; basis with five small spines on anterior margin and without anteroproximal setae, posterior margin with 14 short setae, posterodistal lobe not developed; merus with six clusters of setae and two spines on anterior margin, two spines on posterior margin, one and two spines accompanied by setae on anterodistal and posterodistal corners, respectively; carpus with three groups of spines accompanied by setae on anterior margin, two groups of spines accompanied by setae on posterior margin; propodus with four groups of spines accompanied by setae on anterior margin; dactylus similar to that of pereopod 5.

***Pereopod 7*** (Fig. 5C–E): coxal plate shallowly concave ventrally, four setae on posterior margin; anterior margin of basis with five small spines and without anteroproximal setae, posterior margin with 15 short setae, inner surface near posterodistal corner with four short setae, posterodistal lobe not developed; merus with five clusters of setae and two spine on anterior margin, one spine on posterior margin, two spines accompanied by setae on both anterodistal and posterodistal corners; carpus with three groups of spines accompanied by setae on anterior margin, one spine and one cluster of setae on posterior margin; propodus with four groups of spines accompanied by setae on anterior margin; dactylus similar to those of pereopods 5 and 6.

***Coxal gills*** present on gnathopod 2 and pereopods 3–7.

***Pleonites 1–3*** (Fig. 5K–M): posterodorsal margins of pleonites 1–3 with four, four and five setae, respectively.

***Epimeral plates 1–3*** (Fig. 5H–J): plate 1 with three long setae on anteroventral margin and four short setae on posterior margin; plate 2 with two spines on ventral margin and six short setae on posterior margin; plate 3 with three spines on ventral margin and four short setae on posterior margin.

***Pleopods*** (Fig. 5F, G): peduncle with two retinacula accompanied by one seta; inner ramus slightly longer than outer ramus, both rami fringed with plumose setae.

***Urosomites 1–3*** (Fig. 6F–H): dorsally flat; urosomites 1 and 2 with one-one-one-one spines accompanied by setae on dorsal margins from left to right, respectively; urosomite 3 with two spines accompanied by setae on left and right sides each, and three setae on dorsal margin.

***Uropod 1*** (Fig. 6A): peduncle bearing two basofacial spines, two and three spines on inner and outer margins, respectively, with one spine on both inner and outer distal corners; inner ramus approximately 0.7 × the length of peduncle and almost the same length as outer ramus, with two and one spines on inner and outer margins, respectively; outer ramus with two and three spines on inner and outer margins, respectively; both rami with five distal spines.

***Uropod 2*** (Fig. 6B): peduncle with one spine on inner margin and two spines on outer margin, one spine on both inner and outer distal corners; inner ramus approximately 0.9 × the length of peduncle and 1.3 × as long as outer ramus, with two and one spines on inner and outer margins, respectively; outer ramus with two spines on outer margin; both rami with five distal spines.

***Uropod 3*** (Fig. 6C, D): peduncle with several spines and setae on distal margin; inner ramus approximately 2.0 × as long as peduncle, reaching 0.8 × the length of outer ramus, bearing one distal spine, both inner and outer margins with plumose and simple setae; outer ramus two-articulate, proximal article with three spines on outer margin, bearing three distal spines, both inner and outer margins with plumose and simple setae, terminal article shorter than adjacent spines.

***Telson*** (Fig. 6E): cleft nearly to base, width 0.9 × as long as length, each lobe with one cluster of setae and two single setae on surface, bearing one distal spine accompanied by five setae.

**Descrption of female.** General appearance similar to male. Observed sexual dimorphism as follows:

***Antenna 2*** (Fig. 7A): setae of peduncular articles 4 and 5 longer than those of male, the longest seta on article 4 posterior margin 1.9 × as long as width of article 4, the longest seta of article 5 posterior margin 2.9 × as long as width of article 5.

***Gnathopod 1*** (Fig. 7B, C): palm not as oblique as that of male, with six spines posterior margin, medial palmar spine absent; dactylus not exceeding half of propodus.

***Gnathopod 2*** (Fig. 7D, E): carpus more elongate than that of male, length 1.2 × as long as propodus; palm with two spines on posterodistal corner, medial palmar spine absent.

***Oostegites***: present on gnathopod 2 (Fig. 6D) and pereopods 3–5, with numerous marginal setae.

***Uropod 3*** (Fig. 7F): both rami shorter than those of male, inner ramus 1.3 × as long as peduncle length, and 0.7 × the length of outer ramus.

##### Habitat.

The specimens were collected from a small brook flowing along a cliff on Somaemuldo Island.

##### Molecular analysis.

The COI sequences of *Gammarussomaemulensis* sp. nov. (GenBank accession numbers: ON980527–ON980532) were obtained from six individuals. Additionally, the sequences of *G.wangbangensis* (GenBank accession number: ON980560) and *G.soyoensis* (GenBank accession number: ON980559) were determined in this study. The intraspecific variation of the COI sequence of the new species ranged between 0.0–0.2%. The interspecific variation between new species and the related species ranged between 21.5–26.3% (Table 2).

**Table 2.** A matrix of the uncorrected *p*-distance of the COI sequence of this study.

	Species	1	2	3	4	5	6
**1**	*Gammarussomaemulensis* sp. nov.						
**2**	*G.soyoensis*	0.215					
**3**	*G.sobaegensis*	0.219	0.228				
**4**	*G.baengnyeongensis*	0.243	0.248	0.256			
**5**	*G.gageoensis*	0.251	0.235	0.230	0.210		
**6**	*G.wangbangensis*	0.256	0.245	0.246	0.245	0.281	
**7**	*G.zeongogensis*	0.263	0.217	0.240	0.230	0.206	0.282

##### Remarks.

*Gammarussomaemulensis* sp. nov. is most similar to *G.wangbangensis* Lee & Seo, 1990 in the following features: 1) antenna 2 peduncular article 4 with few clusters of long setae, calceoli absent, 2) pereopods 3 and 4 with long straight setae on posterior margins of merus and carpus, 3) pereopods 5–7 with short setae on posterior margins of basis, and 4) outer ramus of uropod 3 with plumose setae on both margins, setae length of outer margin longer than width of proximal article. However, the new species differs from *G.wangbangensis* in the following features (features of *G.wangbangensis* in parentheses): 1) article 3 of mandibular palp with one group of B-setae (two groups of B-setae), 2) bases of pereopods 6 and 7 without anteroproximal setae (with long anteroproximal setae), 3) terminal article of outer ramus in uropod 3 shorter than adjacent spines (longer than adjacent spines), and 4) anteroventral margin of epimeral plate 1 with three or four setae (six or more setae).

*Gammarussomaemulensis* sp. nov. is also similar to *G.sobaegensis* Uéno, 1966 in the following features: 1) antenna 2 with long setae on peduncular articles, calceoli absent, 2) pereopods 3 and 4 with long straight setae on posterior margins of merus and carpus, and 3) inner ramus of uropod 3 reaching 0.8 × the length of outer ramus. However, the new species can be distinguished from *G.sobaegensis* by the following features (features of *G.sobaegensis* in parentheses): 1) posterior margin of peduncular article 4 in antenna 2 with four clusters of long setae (six or more clusters of long setae), 2) bases of pereopods 6 and 7 without anteroproximal setae (with anteroproximal setae), and 3) outer ramus of uropod 3 with plumose setae on both margins (outer margin without plumose setae).

*Gammarussoyoensis* Lee & Kim, 1980 also share the following features with the new species: 1) antenna 2 calceoli absent and 2) uropod 3 outer margin of outer ramus with plumose setae, terminal article shorter than adjacent spines. However, the new species can be distinguished from *G.soyoensis* by following features (features of *G.soyoensis* in parentheses): 1) male gnathopods 1 and 2 with medial palmar spine, each (without medial palmar spine), 2) setae on posterior margin of merus in pereopod 4 longer than width of merus (shorter than width of merus), and 3) setae on outer margin of outer ramus in uropod 3 longer than width of proximal article (shorter than width of proximal article).

The interspecific variation within the COI sequence ranged from 21.5–26.3% for *G.somaemulensis* sp. nov. and related species (Table 2). Previous studies have reported similar or lower levels of COI sequence divergences among *Gammarus* species. Hou et al. (2009) suggested that the mean inter-specific divergence of the COI sequence among Chinese *Gammarus* species was 21.9%. Copilaș-Ciocianu et al. (2019) reported a 13.3% between *G.hamaticornis* and *G.kischineffensis*. Similarly, Zhang et al. (2022) reported 16.6% difference between *G.zhouqiongi* and *G.takesensis*. Therefore, COI sequence divergence, which is 21.5–26.3% among related species, supports *G.somaemulensis* sp. nov. as a new species.

### ﻿Key to the genus *Gammarus* in South Korea (adult males only)

**Table:** 

1	Antenna 2 caceoli present	**2**
–	Antenna 2 caceoli absent	**4**
2	Posterior margins of pereopod 3 merus and carpus with long straight setae	**3**
–	Posterior margins of pereopod 3 merus and carpus with long curled setae	***G.gageoensis***
3	Length ratio of uropod 3 inner/outer ramus ~ 0.7	***G.baengnyeongensis***
–	Length ratio of uropod 3 inner/outer ramus ~ 0.5	***G.zeongogensis***
4	Inner ramus of uropod 3 with plumose setae on outer margin	**5**
–	Inner ramus of uropod 3 without plumose setae on outer margin	***G.hoonsooi***
5	Peduncular articles 4 and 5 of antenna 2 with long setae	**6**
–	Peduncular articles 4 and 5 of antenna 2 with short setae	***G.galgosensis***
6	Setae on outer margin of outer ramus in uropod 3 short and sparse	**7**
–	Setae on outer margin of outer ramus in uropod 3 long and numerous	**8**
7	Gnathopods 1 and 2 with medial palmar spine on propodus	***G.odaensis***
–	Gnathopods 1 and 2 without medial palmar spine on propodus	***G.soyoensis***
8	Pereopods 5–7 with long setae on posterior margin of basis	**9**
–	Pereopods 5–7 with short setae on posterior margin of basis	**10**
9	Peduncular article 4 of antenna 2 with three or four setal clusters on posterior margin	***G.kyonggiensis***
–	Peduncular article 4 of antenna 2 with seven or eight setal clusters on posterior margin	***G.longisaeta***
10	Outer ramus of uropod 3 with plumose setae on outer margin	**11**
–	Outer ramus of uropod 3 without plumose setae on outer margin	***G.sobaegensis***
11	Terminal article of outer ramus in uropod 3 longer than adjacent spines	***G.wangbangensis***
–	Terminal article of outer ramus in uropod 3 shorter than adjacent spines	***G.somaemulensis* sp. nov.**

## Supplementary Material

XML Treatment for Gammarus somaemulensis
